# Quadrotor-Based Lighthouse Localization with Time-Synchronized Wireless Sensor Nodes and Bearing-Only Measurements

**DOI:** 10.3390/s20143888

**Published:** 2020-07-13

**Authors:** Brian G. Kilberg, Felipe M. R. Campos, Craig B. Schindler, Kristofer S. J. Pister

**Affiliations:** Berkeley Sensor & Actuator Center, Department of Electrical Engineering and Computer Sciences, University of California, Berkeley, CA 94720, USA; fmrcampos@berkeley.edu (F.M.R.C.); craig.schindler@berkeley.edu (C.B.S.); ksjp@berkeley.edu (K.S.J.P.)

**Keywords:** wireless sensor networks, UAVs, indoor localization

## Abstract

Some robotic localization methods, such as ultra wideband localization and lighthouse localization, require external localization infrastructure in order to operate. However, there are situations where this localization infrastructure does not exist in the field, such as robotic exploration tasks. Deploying low power wireless sensor networks (WSNs) as localization infrastructure can potentially solve this problem. In this work, we demonstrate the use of an OpenWSN network of miniaturized low power sensor nodes as localization infrastructure. We demonstrate a quadrotor performing laser-based relative bearing measurements of stationary wireless sensor nodes with known locations and using these measurements to localize itself. These laser-based measurements require little computation on the WSN nodes, and are compatible with state-of-the-art 2 mm × 3 mm monolithic wireless system-on-chips (SoCs). These capabilities were demonstrated on a Crazyflie quadcopter using an Extended Kalman Filter and a network of motes running the OpenWSN wireless sensor network stack. The RMS error for X positioning was 0.57 m and the error for Y positioning was 0.39 m. This is the first use of an OpenWSN sensor network to support robotic localization. Furthermore, simulations show that these same measurements could be used for localizing sensor motes with unknown locations in the future.

## 1. Introduction

Robotic wireless sensor networks (WSNs), defined as networked multi-agent robotic systems intended for sensing the environment [1], have many potential applications, including search and rescue [2], wireless sensor network deployment [3,4], and underground mine exploration [5]. These systems combine the advantages of low-power mesh networking with the mobility afforded by robotic systems. For example, a persistent, low-power WSN could be used as localization reference points for a robot traversing the network area. However, these low-power WSN systems have limited computational resources, which create unique challenges for their use in localization.

Localization is vital for the coordination and control of robotic systems. Additionally, localization is useful for many applications of wireless sensor networks that require geospatial information in addition to sensor data [6,7]. While modern global positioning systems (GPS) function well in most outdoor locations, they do not function reliably in most indoor environments. Some multi-agent robotic control methods also use infrastructure, like VICON, for localization, where high resolution cameras are placed around an environment to triangulate an accurate pose estimate of various objects, which requires significant infrastructure overhead [8] and it is generally not easily scalable. Ultra wideband RF localization is an accurate indoor localization system used in robotic and quadcopter localization, but requires ranging beacon infrastructure [9]. Many robotic localization systems rely on simultaneous localization and mapping (SLAM), which requires enough computational power to process keypoints from images taken of the robot’s surrounding environment [10] and register landmarks globally to perform loop closure. These systems are suitable for robots with enough computational resources, and even then, rely on feature-rich environments. Smaller, low-power, and low-cost robots usually do not have the computational resources to process full images, and they can be spread over a large area, potentially out of range of stationary localization infrastructure.

Many localization methods for resource-constrained robots and low power WSN nodes exist, including ultrasonic, RF, and optical methods. These localization methods use various modalities to obtain measurements of distance, angle of arrival, time of flight, or time delay of arrival. Received signal strength intensity (RSSI), a value that is easy to measure while using RF communication, only provides accuracy on the order of several meters [6,7]. One localization method, lighthouse localization, could be particularly useful for wireless robotic sensor networks due to low computational overhead and spatial resolution in the order of a centimeter [6]. Lighthouse localization [11], intended for smart dust [12], uses laser sweeps to accurately localize computationally resource-constrained sensor motes. The advantage of this system is that the self-localization requires little computation on the sensor motes, because the only calculation required is a time-to-orientation mapping. This localization method also scales well with the number of sensor motes in the system. HTC Vive’s 2015 release of their Lighthouse Base Station, used as the basis for their virtual reality system, led to a resurgence in the use of lighthouse localization in recent years [13]. This HTC Vive lighthouse localization system can provide millimeter-precision 6 degree of freedom pose information to objects with constellations of infrared photodidoes at 60 Hz, while also being low-cost and efficiently scalable for large numbers of nodes [14,15]. Furthermore, lighthouse localization can accurately localize the 2 mm × 3 mm Single Chip μMote (SCμM) wireless SoC [16,17,18]. The 5 mg SCμM requires no external components outside of a battery and antenna, and represents the state of the art in miniaturized wireless sensor nodes. However, lighthouse localization still requires stationary lighthouse beacons to localize sensor motes, which would constrain the spatial range of the robot.

Relative measurements between robots can be used in distributed and cooperative localization methods for teams of robots [19,20,21,22,23]. These methods determine relative positions and orientations between robots using a variety of methods, such as sonar distance estimation for teams of robots [20], cooperative inchworm localization with optical pose estimation [19], and range-only SLAM for multiple micro-air-vehicles [22]. Relative measurements could also be used between wireless sensor nodes and robots for localization. Indeed, numerous relative bearing-only measurements systems have been formulated that allow mobile robots to localize stationary and non-stationary targets [24,25,26]. Bergbreiter et al. [27] demonstrate a particularly applicable system for localizing teams of resource-constrained robots with relative bearing measurements. They use robot-mounted beacon LEDs and fisheye lenses to obtain optical bearing measurements between robots. The system, including a custom integrated circuit that is designed to receive the light signals and a microprocessor for processing, would have a footprint of around 3 cm × 5 cm and it has a light computational load required for localization.

Cooperative localization systems like these have also been demonstrated successfully in some aerial vehicle teams. For example, Roberts et al. [28] develop a 15 cm diameter 3D relative positioning sensor for localizing team of aerial robots. This sensor enables a swarm of flying robots to successfully navigate through an indoor environment while using relative position measurements [29].

We propose a system for drones that provides bearing measurements relative to wireless sensor nodes using laser lighthouse localization and heading measurements that were obtained from an inertial measurement unit (IMU). Similar to the system in Bergbreiter et al. [27], robots carry laser beacon lights to obtain bearing measurements relative to other devices with photodetectors, in this case wireless sensor nodes with known location, or anchor sensor nodes. When compared to the 120 g, 15 cm diameter positioning sensor in [28], the hardware that is required for this system is both smaller and lighter. This system has a low computational load, since the anchor sensor nodes only need to calculate a simple mapping between time and relative bearing. When a quadrotor acts as a lighthouse beacon, it performs a horizontal laser scan by rotating with a mounted vertical planar laser while simultaneously keeping track of its heading angle vs. time in a lookup table. Anchor sensor nodes keep track of pulse timings, as they are struck by this sweep. The lighthouse drone then wirelessly broadcasts its saved heading angle vs. time look up table, and the anchor sensor nodes can interpolate their relative bearing to the lighthouse, and thus determine location information. The lighthouse drone can then receive these bearing-only measurements from the anchor nodes, and incorporate them into its state estimator to improve its position estimate. The low power WSN stack, OpenWSN [30], is used to provide networking communications and a synchronized timebase between the quadrotor and anchor sensor nodes.

**Figure 1:**
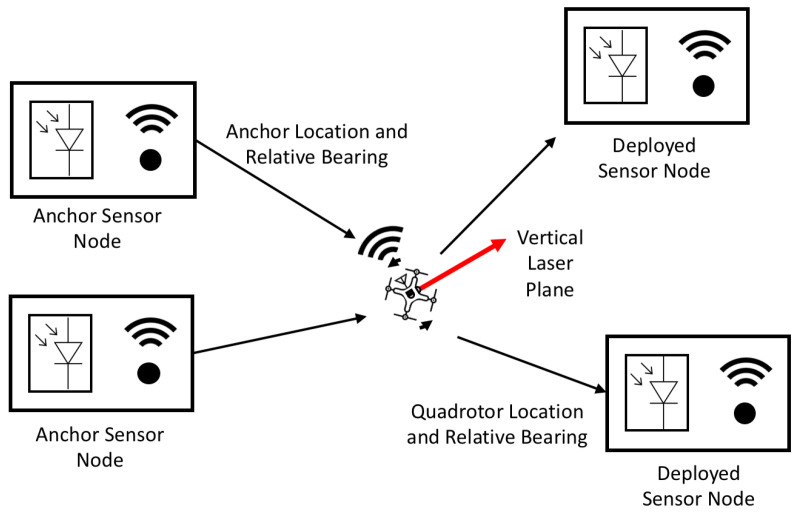
Schematic of proposed distributed lighthouse self-localization. The quadrotor can act as a lighthouse base station by rotating in order to obtain relative bearings between itself and wireless sensor nodes with known locations, or *anchor sensor nodes*. In this system, the quadrotor can localize itself based on bearing measurements relative to these anchor sensor nodes. The quadrotor can also provide relative bearing measurements to recently deployed sensor nodes with unknown locations (*deployed sensor nodes*), which allows them to localize themselves.

This work demonstrates the use of quadrotor-lighthouse bearing measurements, relative to anchor sensor nodes, to estimate the position of a quad rotor (Figure 1). The novel contributions of this work include the use of OpenWSN’s network synchronized timebase to enable relative bearing measurements, the utilization of a quadrotor to perform laser lighthouse scans for obtaining relative bearing measurements to anchor sensor nodes, the modification of OpenWSN to allow network nodes to broadcast relative bearing measurement information, and the implementation of a measurement update to incorporate the relative bearing measurements into the quadrotor’s onborad state estimator. The anchor sensor nodes are based on a miniature 16 mm × 16 mm wireless sensor platform weighing 2 g [31]. With further hardware integration of the photodiode, these sensor nodes could be actively deployed by a quadrotor and then localized by a quadrotor performing lighthouse laser scans. This paper also presents simulations that show that this system should be able to provide relative bearing measurements that can localize wireless sensor nodes with unknown locations, or deployed sensor nodes.

## 2. Materials and Methods

### 2.1. Robotic Lighthouse Localization System

**Figure 2:**
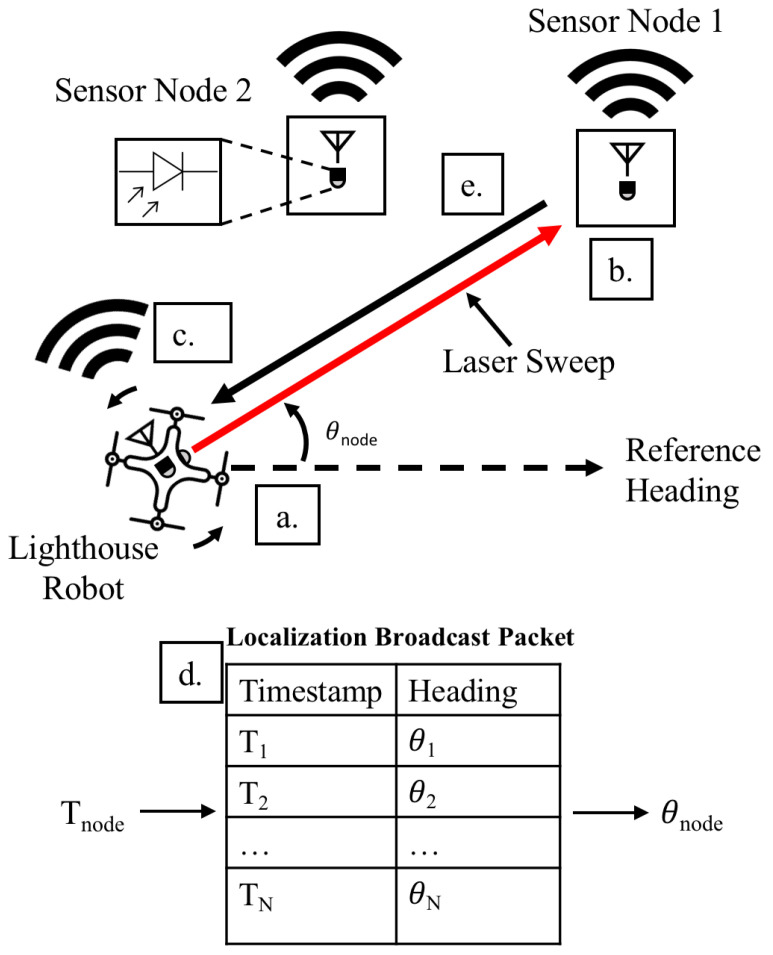
(**a**) The lighthouse quadrotor, with a mounted vertical planar laser, rotates while recording the heading-timestamp information from its attitude estimator. (**b**) Anchor sensor node 1 detects a laser sweep using its photodetector and records the network-synchronized timestamp of the laser detection event at $T_{node}$. (**c**) The lighthouse robot periodically broadcasts its timestamp-orientation mapping to anchor sensor nodes. (**d**) Anchor sensor node 1 uses the received timestamp-orientation broadcast and $T_{node}$ to calculate its bearing $\theta_{node}$ relative to the quadrotor. (**e**) Anchor sensor node 1 sends the relative bearing back to the lighthouse quadrotor, which the quadrotor uses to localize itself.

The quadrotor lighthouse localization system consists of a quadrotor equipped with a low-power IR laser diode and wireless sensor node, and wireless sensor nodes that are equipped with a circuit containing a high radiant sensitivity BPV22NF photodiode with a daylight blocking filter, transimpedance amplifier, passive filter, and gain stage. The wireless sensor node hardware system is comprised of the Micro Inertial Measurement System (MIMSY) [31], a 16 mm × 16 mm wireless sensor node containing a Cortex-M3 microprocessor, 9-axis IMU, and 802.15.4 transceiver. The quadrotor’s 1 mW laser diode is equipped with line-generating optics, which produces a vertical planar laser beam. These components are used to produce the lighthouse sweep, record the lighthouse bearing measurements, and transmit the timestamp-heading tuples (Figure 2) (drone icon adapted from icons made by Freepik from www.flaticon.com).

Figure 2 illustrates the operation of the quadrotor lighthouse localization system. As the lighthouse robot rotates, it records its headings and broadcasts them, along with corresponding network-synchronized timestamps, to every anchor sensor node in range. When the anchor sensor nodes detect a lighthouse laser pulse, they compare the laser pulse time to the timestamped heading broadcast in order to determine their bearing relative to the lighthouse robot. These bearings, along with the corresponding anchor sensor node locations, are transmitted back to the quadrotor, which can use them to localize itself.

**Figure 3:**
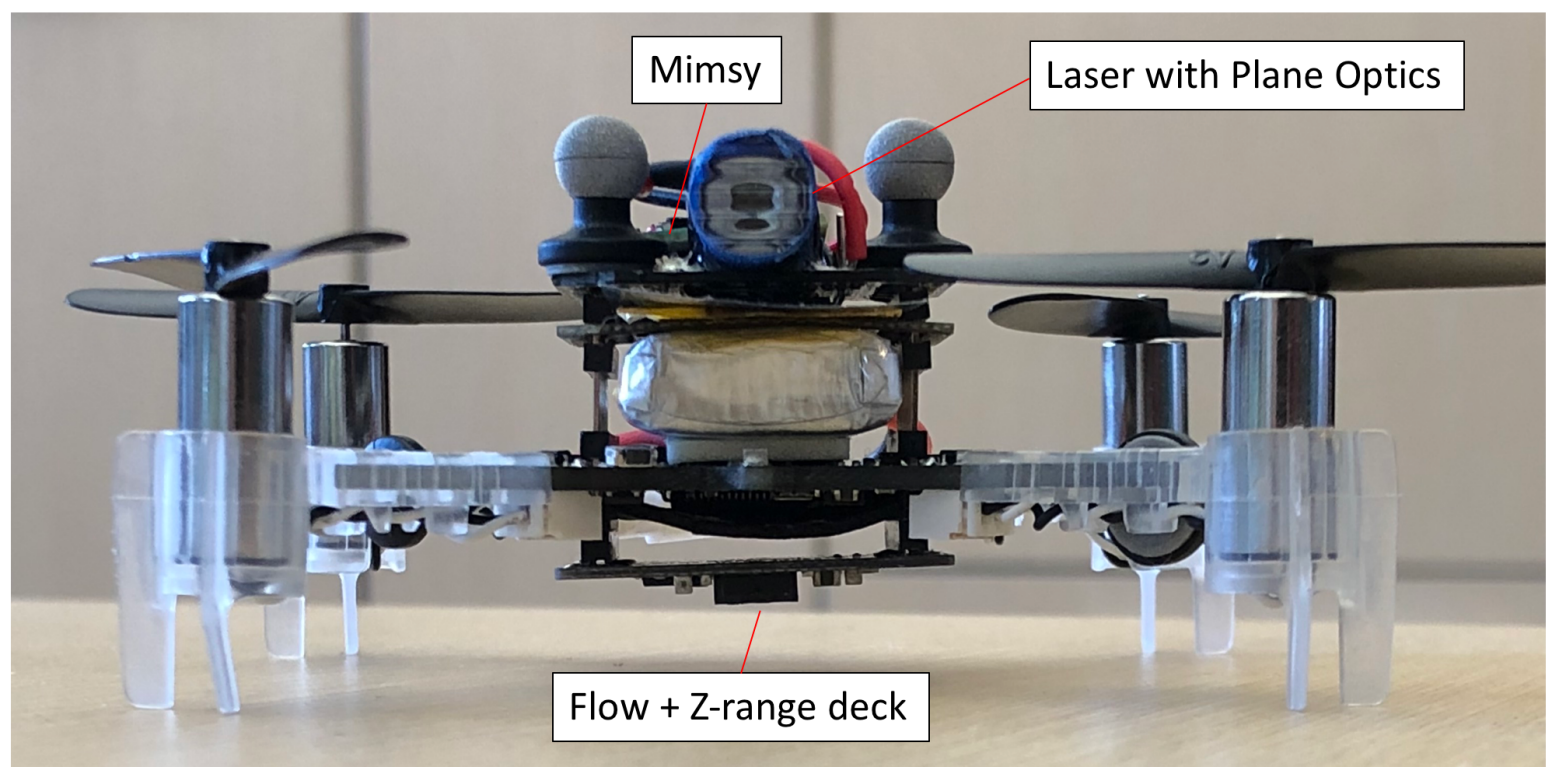
Crazyflie minidrone platform with a Micro Inertial Measurement System (MIMSY), 850 nm laser, plane-generating optics, and an optical flow deck.

**Figure 4:**
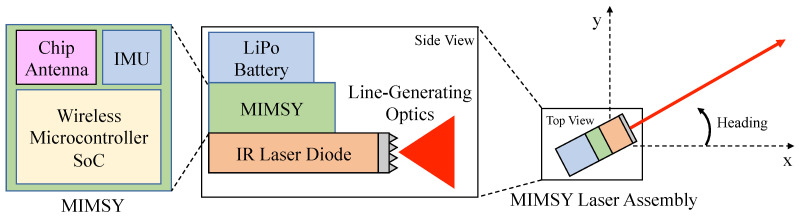
System Block Diagram.

The wireless sensor hardware is integrated into a Bitcraze Crazyflie 2.0 quadrotor using a UART interface. The Crazyflie 2.0 performs all of the real-time state estimation calculations and periodically updates the MIMSY with its X position, Y position, and yaw estimates via UART at 40 Hz. These estimates are obtained via its on-board state estimator, which is further described in the State Estimation section of this paper. The MIMSY communicates any lighthouse measurements that it receives to the quadrotor using this UART interface. Figure 3 shows the full MIMSY, laser and Crazyflie system. Figure 4 shows the block diagram of this MIMSY laser assembly. The Crazyflie system also includes an optical flow deck, which contains an optical flow sensor and z-ranging infrared sensor. These provide velocity and *Z* measurements to the quadrotor. An Extended Kalman Filter (EKF) included in the Crazyflie firmware is used to provide heading information to the MIMSY for the lighthouse measurements. The 9 × 9 cm${}^{2}$ Crazyflie has a maximum takeoff weight of 42 g, contains a 240 mAh LiPo battery, and uses 4 × 7 mm coreless DC motors as actuators.

**Figure 5:**
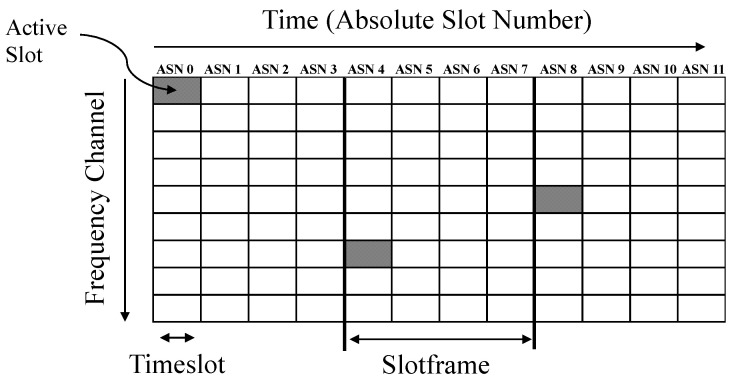
Time-synchronized channel hopping (TSCH) matrix for OpenWSN. Timeslots in the TSCH matrix correspond to points in time and frequency and can be scheduled as active and inactive. Active slots can be used for transmitting or receiving packets, while inactive slots are used for saving power. All the motes in a TSCH network must maintain a common synchronized sense of time in order for this network architecture to operate. The absolute slot number (ASN) reflects the current network-wide time in units of timeslot, which are 10 ms long in this application. This universal timebase enables infrastructure-free lighthouse localization.

Synchronized timekeeping is imperative for this proposed quadrotor lighthouse method to function; every robot in the network must have a synchronized sense of time in order to calculate their relative bearings to the lighthouse robot. Fortunately, many wireless sensor network architectures already maintain a synchronized network-wide timebase during normal operation [32,33]. Specifically, IEEE 802.15.4E networks use Time-Synchronized Channel Hopping (TSCH), which improves network reliability and reduces power consumption [32,34]. A synchronized sense of time is maintained between all sensor nodes in the network and it is denoted by the absolute slot number (ASN), which indicates the current timeslot number of the network. Sensor nodes in the network communicate, wake-up, or sleep, depending on the current timeslot and where it resides within the network schedule. They follow a synchronized schedule that dictates which channel the nodes’ radios should be using at which time subdivision. This schedule is called the TSCH matrix and it is illustrated in Figure 5. This figure also illustrates the network-synchronized ASN that is used for the relative bearing measurements. The ASN only provides time resolution according the timeslot length, which is 10 ms in our network implementation; we improved this resolution by using the sensor node’s onboard crystal timers to obtain 30.5 μs resolution when timing lighthouse-related events.

**Figure 6:**
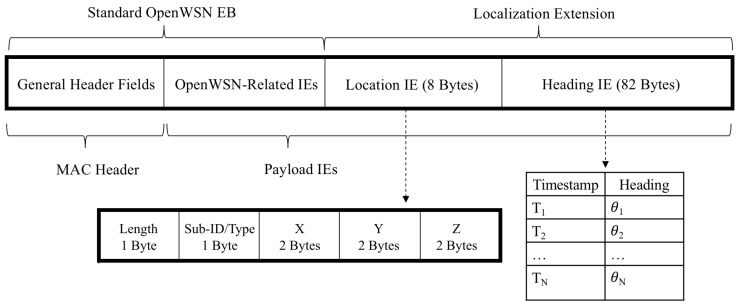
OpenWSN sensor nodes periodically broadcast packets, called enhanced beacons (EBs), which contain network-related data. EBs contain Information Elements (IEs), which are containers used to store specific types of payload. The lighthouse sensor node uses EBs to broadcast time-heading tuples.

The wireless sensor network implementation we used was OpenWSN, which is a full-stack, standards-compliant implementation of a wireless sensor network based on the IEEE 802.15.4E standard [30]. OpenWSN can run on low-power, resource-constrained devices and provides routing, scheduling, transport layer, and internet integration. In addition to a synchronized timebase, OpenWSN provides peer-to-peer communication in the form of Enhanced Beacons. Enhanced Beacons (EBs) are periodically broadcast by all sensor node in an OpenWSN network and they are used to advertise the network, so new nodes can join [35]. Information Elements (IEs) are information containers within EBs that can carry various types of network-related information. For use in the distributed lighthouse system, we create location and time-heading tuple IEs, which enable the transmission of the information necessary for sensor nodes to perform localization from a laser pulse. Figure 6 shows the formatting of the EB packet that was broadcast by beacon robots. These beacons were redundantly retransmitted three times in order to improve the reliability of the communication, which leveraged reliability improvements possible with TSCH.

**Figure 7:**
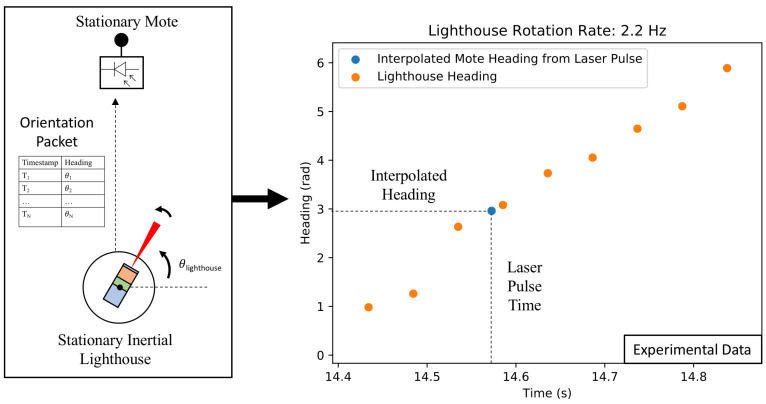
Calculation of bearing from laser pulse timing. Once a node detects a laser pulse and receives a localization EB, it can calculate its bearing relative to the lighthouse node by interpolating the surrounding lighthouse time-heading tuples and looking up the laser pulse time. This data was obtained during an experiment with a fixed-position lighthouse sensor node rotating and a stationary self-localizing sensor node. This heading data were produced with the on-board magnetometer on a MIMSY that was located indoors.

When a sensor node detects a quadrotor laser sweep pulse with its photodiode, it records the pulse’s timestamp. It then waits until it receives a localization EB. Once a localization EB and laser pulse have been received, the sensor calculates the direction that the quadrotor’s laser was pointing when the laser struck the photodiode, providing the sensor node’s bearing relative to the quadrotor. During the calculation procedure, the sensor node searches the localization EB tuples until it finds a time-heading tuple before and a tuple after the recorded laser pulse time. The sensor node then linearly interpolates between these two points and calculates the heading that corresponds to the laser pulse time (Figure 7).

In this work, the wireless sensor nodes used were MIMSYs; however, the underlying robotic lighthouse system is compatible with the Single-Chip Mote (SCM), which is a monolithic wireless system-on-a-chip (SoC) that requires no external components [16,17]. This mote contains an IEEE 802.15.4 radio, allowing it to support OpenWSN, which provides the networking backbone for this measurement system. Furthermore, the SCM contains an optical receiver that has been shown to provide accurate localization performance in a commercial HTC lighthouse system [17]. This compatibility will allow for cubic millimeter sensor motes to act as localization anchors and eventually be localized through the robotic lighthouse measurement system.

### 2.2. Wireless Reliability

**Figure 8:**
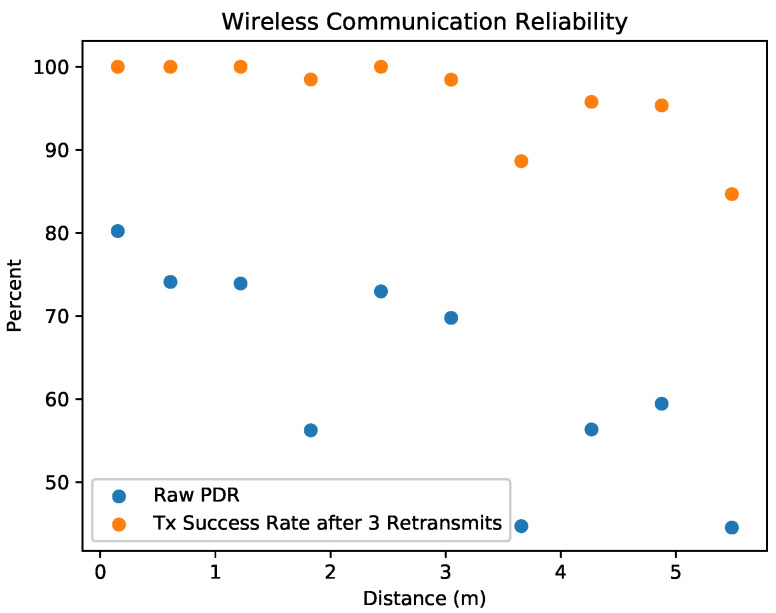
Reliability of robot to anchor beacon broadcasts within OpenWSN. Each broadcast from robot to anchor consisted of three redundant retransmissions of the broadcast’s packet. Packet delivery rate (PDR) was calculated as the ratio of all packets (including redundant retransmissions) that are successfully received by the anchor, which is also approximately the success rate of a broadcast not using redundant transmissions. Transmission success rate was the percent of broadcasts where at least one of the three retransmitted packets were received. RF interference within the experiment space decreased the packet, which is evident in the low PDRs at short distances. Retransmitting packets three times improved the success rate of the transmissions.

The proposed localization framework is dependent on reliable communications, especially with its relatively slow measurement rate. As the measurement communication process requires a successful round trip communication between the lighthouse robot and the anchor points, which consists of two successful transmissions. For example, if the packet delivery ratio (PDR), the ratio of transmitted packets that are successfully received, of the link is 80%, then only 64% of round-trip measurement communications will be successful, effectively reducing the update rate of measurements in the system. Unfortunately, the low power 2.4 GHz radios and suboptimal chip antennas used in this hardware system and many other robotics systems are susceptible to path loss, interference, and multipath. Indeed, PDR measurement experiments for our hardware taken in the experiment volume were not optimistic (Figure 8). For this specific hardware, the PDR up to three meters was roughly 75%, while the PDR at distances between three and six meters was 45% and 65%.

Fortunately, wireless sensor networks use similar radio hardware that yields lossy communications, and significant effort has gone into developing methods to cope with lossy communications in wireless sensor networks. Time-synchronized channel hopping (TSCH) [32], used in some wireless sensor networks, helps networks to overcome multipath effects and in-channel interference that reduce reliability. Indeed, some industrial wireless sensor networks using TSCH and packet retransmissions can obtain up to nine nines of reliability (99.9999999% of transmissions completed successfully) [36,37].

Packet retransmissions with TSCH were used in order to overcome the low PDRs inherent in the low power radios and the experiment setting. Without TSCH, rapidly retransmitting packets is ineffective at improving reliability, because retransmissions occur on the same channel frequency, and any disturbance is also likely to affect retransmissions. Conversely, retransmitting rapidly on different channels can provide greater reliability because each channel is independent [38,39]. In this work, each broadcast consisted of three redundant transmissions of the same data packet. With the timeslot of 10 ms in this network, each redundant packet should be sent 10 ms after the previous transmission. Figure 8 shows this method providing >98% reliability up to distances of three meters, and 80–95% reliability in distance between three and six meters. This reliability improvement likely would not have been possible if used in a non-TSCH network.

### 2.3. State Estimation

#### Quadrotor Localization with Bearing Measurements

State estimation and sensor fusion is required in this system in order to provide timely state information for the quadrotor. The estimator used in this work is based on the Extended Kalman Filter (EKF) derived in [9,40] and implemented by Bitcraze in the Crazyflie firmware, which utilizes IMU data for state propagation and can incorporate a variety of measurement updates. The robotic lighthouse measurements were incorporated into the state estimator as measurements. Each measurement contained the location of the sensor node anchor point the measurement came from and the relative bearing measurement to that anchor point.

The Crazyflie estimator has nine states (Equation (1)), where x,y,z denote position, vx,vy,vz denote velocity and D0,D1, and D2 denote attitude error [9,40]. Attitude is stored in a separate reference attitude, which is periodically updated using the attitude error states.

(1) $$\vec{x}={\left[\begin{array}{c} x\;y\;z\;vx\;vy\;vz\;D0\;D1\;D2 \end{array}\right]}^{T}$$

The measurement model for a lighthouse relative bearing measurement between a rotating quadrotor, with two-dimensional (2D) location (x,y), and a stationary anchor point, with 2D location $\left(x_{a},y_{a}\right)$, at timestep *k* is shown in Equation (2), where *w* represents gaussian noise. The current state prediction at timestep *k* is denoted by ${\hat{x}}_{p}\left(k\right)$. The linearization, *H*, of this measurement model with respect to the state model is shown in Equations (3) and (4), where the only nonzero entries correspond to the *x* and *y* states. In Equation (4), *R* is the distance between the anchor point and the quadrotor’s estimated X,Y locations, and θ is the predicted bearing measurement. This equation *H* is added to the standard EKF measurement update equations in [9,40].

(2) $$h_{k}\left({\hat{x}}_{p}\left(k\right),w\left(k\right)\right)=atan2\left(\frac{y_{a}-y\left(k\right)}{x_{a}-x\left(k\right)}\right)+w$$

(3) $$H=\frac{\partial h_{k}\left({\hat{x}}_{p}\left(k\right),0\right)}{\partial \vec{x}}$$

(4) $$H=\frac{1}{R}\left[\begin{array}{ccccc} sin(\theta ) & -cos(\theta ) & 0 & \ldots & 0 \end{array}\right]$$

(5) $$R=\sqrt{{\left(x_{a}-{\hat{x}}_{p}\right)}^{2}+{\left(y_{a}-{\hat{y}}_{p}\right)}^{2}}$$

(6) $$\theta =h_{k}\left({\hat{x}}_{p}\left(k\right),0\right)=atan2\left(\frac{y_{a}-y\left(k\right)}{x_{a}-x\left(k\right)}\right)$$

This estimate was incorporated into the Crazyflie’s EKF, and the measurement update was performed each time a quadrotor lighthouse measurement was detected. All of the state estimation was computed onboard the Crazyflie’s microprocessor.

## 3. Results

**Figure 9:**
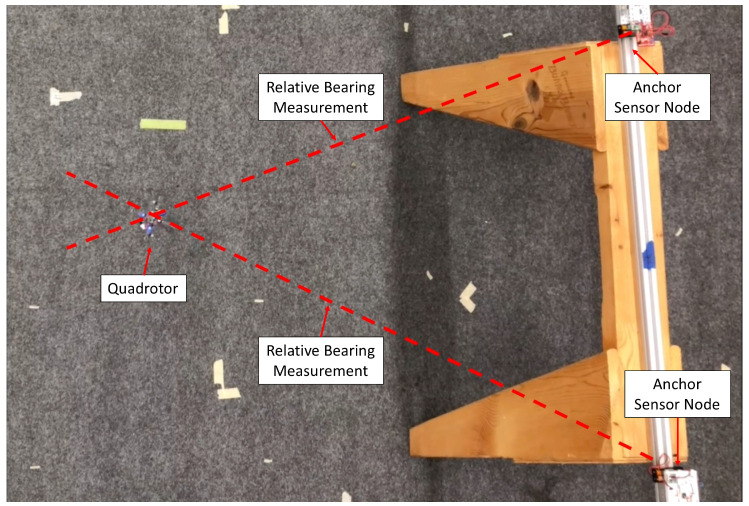
Setup of localization experiment. This is a top-down view of the experiment, and the motion capture cameras are not visible.

The experiments were performed in a motion capture room using an OptiTrack system for capturing ground truth information with sub-millimeter accuracy. In this experiment, the Crazyflie took off and performed rotations using open loop control. Figure 9 shows this experimental setup in the motion capture room. Estimation data was streamed off the quad using the Crazyflie’s logging capabilities and radio. Two wireless sensor node anchors with known positions were present during the experiment and they were used to localize the quad. Before the quad took off, its state estimator was reset and its state variance was set to maximum.

**Figure 10:**
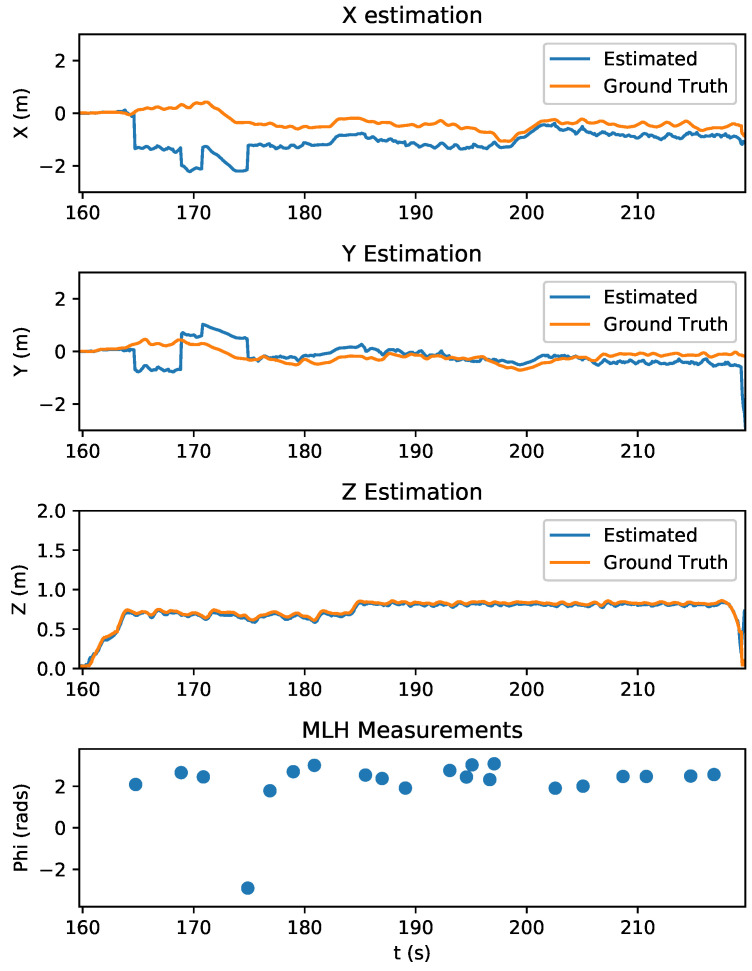
Results of X, Y, and Z state estimates during a quadrotor lighthouse experiment. Initial lighthouse bearing measurements were heavily weighted due to high initial state variance (see Figure 12). This is illustrated by the large discontinuities in tracking between 165 and 175 s, which correspond to lighthouse measurement updates. As the Crazyflie received more measurements from the sensor nodes, the state variance decreased (Figure 12), the measurements were less heavily weighted, and the estimator started to recover and track with less error. The bottom graph shows the received lighthouse measurements. The Z state estimation was very accurate and can be attributed to Bitcraze’s stock state estimation with optical flow deck, which includes an infrared range sensor. The bottom graph shows the quadrotor MIMSY lighthouse measurements (MLH) received by the quadrotor.

**Figure 11:**
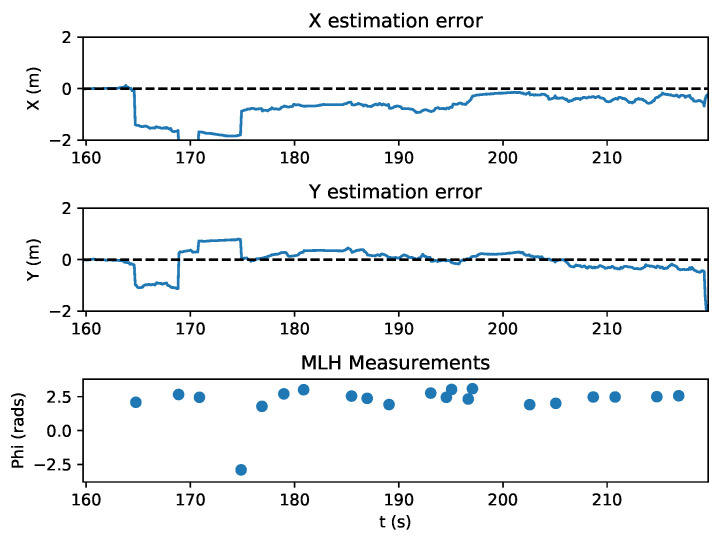
Estimation error during the quadrotor lighthouse localization experiment. The quadrotor is performing rotations and receiving bearing measurements from sensor nodes.

**Figure 12:**
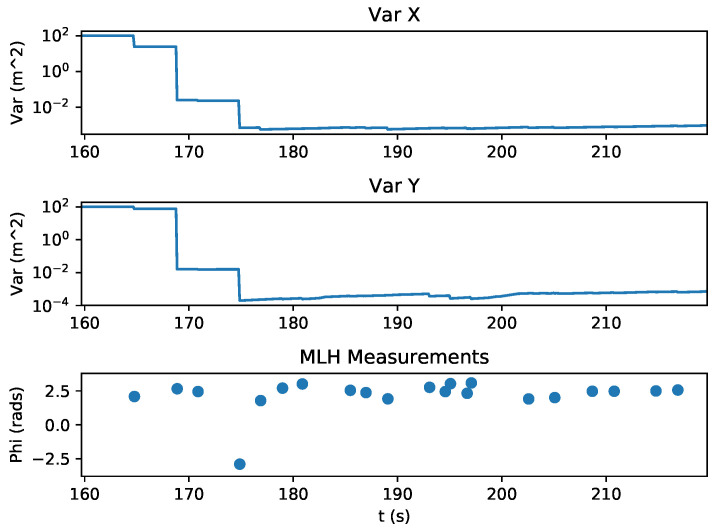
Estimator Variance throughout the experiment. The MIMSY lighthouse measurements (MLH) decreased the estimated variance of the x and y states, as expected.

**Figure 13:**
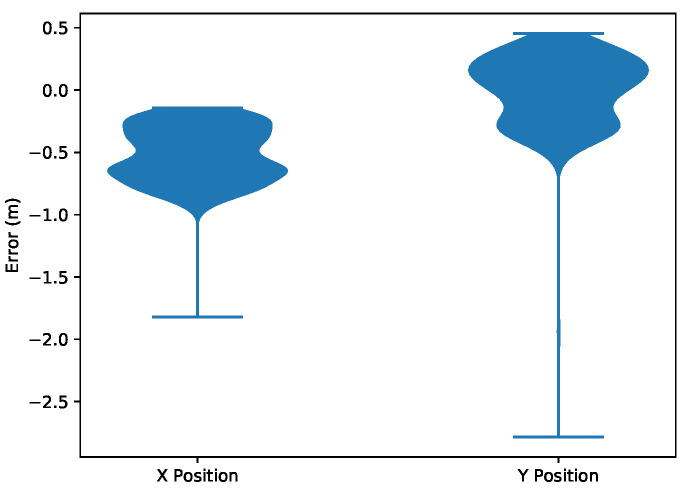
Violin plots showing the error distribution of the X and Y position state estimation.

**Figure 14:**
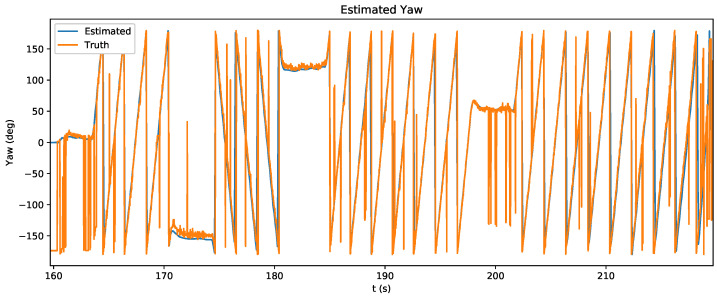
Yaw estimation during experiment. Pose was estimated using the stock Crazyflie EKF algorithm, which relies on gyroscope measurement data.

**Figure 15:**
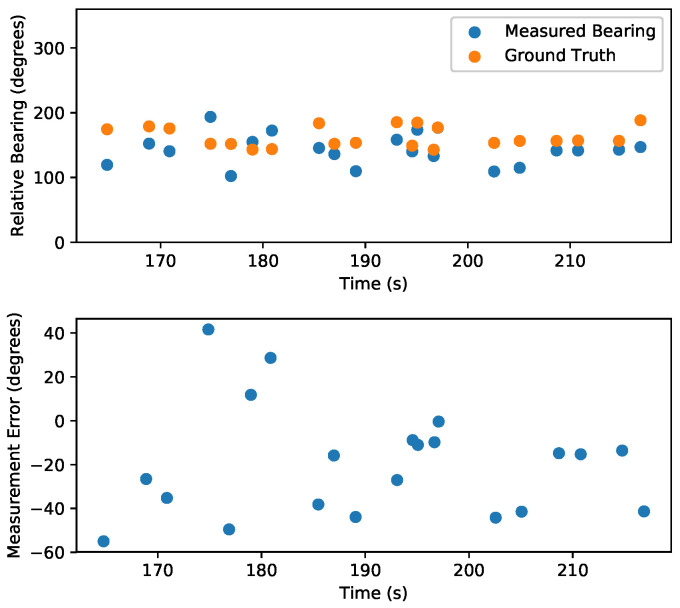
Measurement error during quadrotor lighthouse experiment. Top: bearing measurements compared to actual relative bearings determined by ground truth data. Bottom: measurement error. The error bias (mean error) was −19.5 degrees, and the standard deviation was 24.7 degrees. This error is larger than expected, given the accuracy of the yaw estimates. A likely cause of error could be timing errors introduce by the firmware interface between the wireless sensor node performing measurements and the Crazyflie.

The state estimation of the quadrotor during this experiment is shown in Figure 10. With the measurement variance set to 0.0025 radians, the first relative bearing measurement of the experiment had a large effect on the X and Y states, which had high initial state variance of 100 m${}^{2}$. Subsequent measurements helped the estimator to recover and reduce its error, which is shown in Figure 11. Figure 12 shows the estimated X and Y variance, as calculated by the EKF, throughout the experiment. Performance could have also been affected by a firmware bug where Equation (4) was given the actual bearing measurement instead of the predicted bearing measurement $\theta =h_{k}\left({\hat{x}}_{p}\left(k\right),0\right)$. The RMS error for X position was 0.57 m and the RMS error for Y position was 0.39 m. This error was measured after the filter converged (at 175 s), which was determined by the point in time where filter variance converged, as seen in Figure 12. Violin plots showing the distributions of the errors are shown in Figure 13. The lighthouse bearing measurements clearly reduce the estimator’s variance estimate throughout the experiment. The yaw estimates broadcast by the rotating drone were obtained from the Crazyflie’s attitude estimates, which were based off of the gyroscope measurements from its IMU (Figure 14). Over the timeframe of this experiment, gyroscope drift accumulated to 20 degrees of error near the end of the quadrotor’s flight. For longer experiments or missions, magnetometer data could be incorporated to reduce gyroscope drift. Some anchor sensor nodes could be reserved for providing relative bearing measurements that would be used for updating the heading estimate of the quadrotor rather than the position estimate. The measurement error is shown in Figure 15, which compares the measured relative bearing to the actual bearing between the anchor and quadrotor, as calculated with motion capture ground truth data.

### Wireless Sensor Node Localization Simulation

Currently, this robotic lighthouse localization system can provide bearing measurements for a rotating quadrotor acting as a lighthouse. This same quadrotor should be able to provide bearing only measurements to wireless sensor nodes that have unknown locations, as has been shown in the literature [24,25,26]. This capability would be useful for situations where a wireless sensor network is deployed and locations cannot be easily surveyed, like if many miniature wireless sensor nodes were dispersed by a UAV throughout an area. For example, in Mehta et al. [41], a micro-air-vehicle (MAV) is used to deploy a “breadcrumb trail” of wireless sensor nodes that join a wireless mesh network, where the MAV is effectively deploying its own networking infrastructure. These nodes were then used as wireless infrastructure to relay commands to the MAV and transmit video signal from the MAV to a base station. With the presented robotic lighthouse localization system, these node could also act as localization infrastructure for the MAV. The combination of wireless mesh networks and robotic lighthouse localization could enable a MAV to self-deploy its own localization and communication infrastructure by dropping wireless nodes as it travels through a novel area.

**Figure 16:**
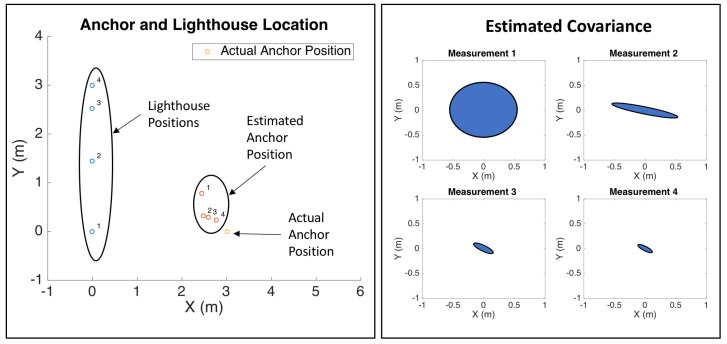
Simulation of wireless sensor node localization using lighthouse bearing measurements from a rotating quadrotor that is also moving translationally. In this simulation, the sensor node doesn’t know its location and is estimating its location from bearing measurements from the quadrotor lighthouse. As the anchor sensor mote receives more measurements, its location estimate improves, and estimated variance decreases.

We formulated the sensor node state estimation model is an EKF that uses the nonzero portion of the measurement model in Equation (4), modified with reversed signs as the Jacobian is with respect to the two anchor state $\vec{x_{a}}=\left[x_{a}\;y_{a}\right]$. Because the mote is stationary, there is no state propagation step in the estimator. The EKF allows the sensor node to estimate its state variance, which it could use to determine when its location estimate is good enough to be used as a localization reference node. Figure 16 shows the results of a simulation of a rotating lighthouse robot providing bearing-only measurements to an unknown wireless sensor anchor node. Future work will focus on verifying this simulation in experiments, and demonstrating this localization ability.

## 4. Discussion

The goal of this work was to implement a bearing-only lighthouse localization that could be used by a robot to localize itself from wireless anchor sensor nodes, and eventually be used to localize other deployed sensor nodes. We achieved this by using attitude estimates from a quadrotor’s EKF, the time-synchronization and peer-to-peer communication provided by OpenWSN, low-power IR lasers, photodiodes, and drone maneuvers. We demonstrated the use of these relative bearing measurements in a Crazyflie’s onboard EKF to estimate position, and performed simulations that show that this system should be able to localize deployed sensor nodes with an unknown location. The RMS errors for the X and Y position state estimates were 0.56 m and 0.39 m, respectively. This is a reduction in performance as compared to the system in [29] with around 10–15 cm of error at comparable distances, but the hardware required for our system was significantly smaller and lighter, <5 g and 2 cm × 2 cm versus 150 g and 15 cm diameter. The laser scan produced by the quadrotor is also detectable by a 5 mg, 2 mm × 3 mm single chip mote, which means that this localization method could be used in the future for localizing a network of highly miniaturized wireless sensor nodes. Future work will focus on experimentally demonstrating the localization of wireless sensor nodes with unknown position using this measurement system, extending this method to localizing other quadrotors, and incorporating multiple lighthouse robots into the system.

## References

[1] Ghosh P., Gasparri A., Jin J., Krishnamachari B. (2017). Robotic Wireless Sensor Networks. arXiv.

[2] Penders J., Alboul L., Witkowski U., Naghsh A., Saez-Pons J., Herbrechtsmeier S., El-Habbal M. (2011). A robot swarm assisting a human fire-fighter. Adv. Rob..

[3] Batalin M.A., Sukhatme G.S. (2004). Coverage, exploration and deployment by a mobile robot and communication network. Telecommun. Syst..

[4] Correll N., Bachrach J., Vickery D., Rus D. (2009). Ad-hoc wireless network coverage with networked robots that cannot localize. Proceedings of the 2009 IEEE International Conference on Robotics and Automation.

[5] Weiss M.D., Peak J., Schwengler T. (2008). A Statistical Radio Range Model for a Robot MANET in a Subterranean Mine. IEEE Trans. Veh. Technol..

[6] Iliev N., Paprotny I. (2015). Review and comparison of spatial localization methods for low-power wireless sensor networks. IEEE Sens. J..

[7] Mao G., Fidan B., Anderson B.D. (2007). Wireless sensor network localization techniques. Comput. Netw..

[8] Merriaux P., Dupuis Y., Boutteau R., Vasseur P., Savatier X. (2017). A study of vicon system positioning performance. Sensors.

[9] Mueller M.W., Hamer M., D’Andrea R. (2015). Fusing ultra-wideband range measurements with accelerometers and rate gyroscopes for quadrocopter state estimation. Proceedings of the 2015 IEEE International Conference on Robotics and Automation (ICRA).

[10] Durrant-Whyte H., Bailey T. (2006). Simultaneous localization and mapping: Part I. IEEE Rob. Autom. Mag..

[11] Römer K. (2003). The lighthouse location system for smart dust. Proceedings of the 1st International Conference on Mobile Systems, Applications and Services.

[12] Warneke B., Last M., Liebowitz B., Pister K.S. (2001). Smart dust: Communicating with a cubic-millimeter computer. Computer.

[13] Campos F.M.R., Schindler C.B., Kilberg B.G., Pister K.S.J. (2020). Lighthouse Localization of Wireless Sensor Networks for Latency-Bounded, High-Reliability Industrial Automation Tasks. Proceedings of the 2020 16th IEEE International Conference on Factory Communication Systems (WFCS).

[14] Borges M., Symington A.C., Coltin B., Smith T., Ventura R. (2018). HTC Vive: Analysis and Accuracy Improvement. Proceedings of the 2018 IEEE/RSJ International Conference on Intelligent Robots and Systems (IROS).

[15] Yang Y., Weng D., Li D., Xun H. (2017). An Improved Method of Pose Estimation for Lighthouse Base Station Extension. Sensors.

[16] Maksimovic F., Wheeler B., Burnett D.C., Khan O., Mesri S., Suciu I., Lee L., Moreno A., Sundararajan A., Zhou B. (2019). A Crystal-Free Single-Chip Micro Mote with Integrated 802.15. 4 Compatible Transceiver, sub-mW BLE Compatible Beacon Transmitter, and Cortex M0. 2019 Symposium on VLSI Circuits.

[17] Wheeler B., Ng A., Kilberg B., Maksimovic F., Pister K.S.J. A Low-Power Optical Receiver for Contact-free Programming and 3D Localization of Autonomous Microsystems. Proceedings of the IEEE UEMCON.

[18] Kilberg B.G., Campos F.M.R., Maksimovic F., Pister K.S.J. (2020). Accurate 3D Lighthouse Localization of a Low-Power Crystal-Free Single Chip Mote. Solid-State Sensors, Actuatorsand Microsystems Workshop (Hilton Head).

[19] Nemsick B.E., Buchan A.D., Nagabandi A., Fearing R.S., Zakhor A. (2017). Cooperative inchworm localization with a low cost team. Proceedings of the 2017 IEEE International Conference on Robotics and Automation (ICRA).

[20] Grabowski R., Navarro-Serment L.E., Paredis C.J., Khosla P.K. (2000). Heterogeneous teams of modular robots for mapping and exploration. Autonom. Rob..

[21] Roumeliotis S.I., Bekey G.A. (2002). Distributed multirobot localization. IEEE Trans. Rob. Autom..

[22] Fabresse F.R., Caballero F., Ollero A. (2015). Decentralized simultaneous localization and mapping for multiple aerial vehicles using range-only sensors. Proceedings of the 2015 IEEE International Conference on Robotics and Automation (ICRA).

[23] Wanasinghe T.R., Mann G.K.I., Gosine R.G. (2014). Distributed collaborative localization for a heterogeneous multi-robot system. Proceedings of the 2014 IEEE 27th Canadian Conference on Electrical and Computer Engineering (CCECE).

[24] Hoffmann G.M., Tomlin C.J. (2010). Mobile Sensor Network Control Using Mutual Information Methods and Particle Filters. IEEE Trans. Autom. Control.

[25] Hammel S., Liu P., Hilliard E., Gong K. (1989). Optimal observer motion for localization with bearing measurements. Comput. Math. Appl..

[26] Grocholsky B., Makarenko A., Durrant-Whyte H. (2003). Information-theoretic coordinated control of multiple sensor platforms. Proceedings of the 2003 IEEE International Conference on Robotics and Automation (Cat. No.03CH37422).

[27] Bergbreiter S., Mehta A., Pister K.S. PhotoBeacon: Design of an optical system for localization and communication in multi-robot systems. Proceedings of the ROBOCOMM 2007.

[28] Roberts J.F., Stirling T., Zufferey J.C., Floreano D. (2012). 3-D relative positioning sensor for indoor flying robots. Autonom. Rob..

[29] Stirling T., Roberts J., Zufferey J., Floreano D. (2012). Indoor navigation with a swarm of flying robots. Proceedings of the 2012 IEEE International Conference on Robotics and Automation.

[30] Watteyne T., Vilajosana X., Kerkez B., Chraim F., Weekly K., Wang Q., Glaser S., Pister K. (2012). OpenWSN: A standards-based low-power wireless development environment. Trans. Emerg. Telecommun. Technol..

[31] Schindler C.B., Drew D.S., Kilberg B.G., Campos F.M., Yanase S., Pister K.S. (2019). MIMSY: The Micro Inertial Measurement System for the Internet of Things. Proceedings of the 2019 IEEE 5th World Forum on Internet of Things (WF-IoT).

[32] Pister K., Doherty L. (2008). TSMP: Time synchronized mesh protocol. IASTED Distrib. Sens. Netw..

[33] Watteyne T., Mehta A., Pister K. (2009). Reliability through frequency diversity: Why channel hopping makes sense. Proceedings of the 6th ACM Symposium on Performance Evaluation of Wireless Ad Hoc, Sensor, and Ubiquitous Networks.

[34] IEEE 802.15.4e-2012—IEEE Standard for Local and Metropolitan Area Networks–Part 15.4: Low-Rate Wireless Personal Area Networks (LR-WPANs) Amendment 1: MAC Sublayer. https://ieeexplore.ieee.org/document/6012487.

[35] Vilajosana X., Pister K., Watteyne T. (2017). Minimal IPv6 over the TSCH Mode of IEEE 802.15.4e (6TiSCH) Configuration. RFC 8180. https://tools.ietf.org/html/rfc8180.

[36] Luvisotto M., Pang Z., Dzung D. (2017). Ultra High Performance Wireless Control for Critical Applications: Challenges and Directions. IEEE Trans. Ind. Inf..

[37] Swamy V.N., Suri S., Rigge P., Weiner M., Ranade G., Sahai A., Nikolic B. (2015). Cooperative communication for high-reliability low-latency wireless control. IEEE Int. Conf. Commun..

[38] Kilberg B., Schindler C.B., Sundararajan A., Yang A., Pister K.S.J. (2018). Experimental Evaluation of Low-Latency Diversity Modes in IEEE 802.15.4 Networks. Proceedings of the 2018 IEEE 23rd International Conference on Emerging Technologies and Factory Automation (ETFA).

[39] Schindler C.B., Watteyne T., Vilajosana X., Pister K.S.J. (2017). Implementation and characterization of a multi-hop 6TiSCH network for experimental feedback control of an inverted pendulum. Proceedings of the 2017 15th International Symposium on Modeling and Optimization in Mobile, Ad Hoc, and Wireless Networks, WiOpt 2017.

[40] Mueller M.W., Hehn M., D’Andrea R. (2016). Covariance correction step for kalman filtering with an attitude. J. Guid. Control Dyn..

[41] Mehta A., Kerkez B., Glaser S.D., Pister K.S. (2012). TDMA-based dual-mode communication for mobile wireless sensor networks. Sensors.

